# LncRNA LINC01569 promotes M2 macrophage polarization to accelerate hypopharyngeal carcinoma progression through the miR-193a-5p/FADS1 signaling axis

**DOI:** 10.7150/jca.83466

**Published:** 2023-06-04

**Authors:** Qilin Gong, Huaying Li, Jintian Song, Chang Lin

**Affiliations:** 1 Department of Otolaryngology Head and Neck Surgery, The First Affiliated Hospital of Fujian Medical University, Fuzhou 350004, Fujian Province, China.; 2 Department of Head and neck surgery, Clinical Oncology School of Fujian Medical University, Fujian Cancer Hospital, Fuzhou 350014, Fujian Province, China.; 3 Fujian Key Laboratory of Rehabilitation Technology. Fuzhou 350003, Fujian Province, China.; 4 Gastrointestinal Endoscopy Department, Rehabilitation Hospital Affiliated to Fujian University of Traditional Chinese Medicine, Fuzhou 350003, Fujian Province, China.; 5 Department of Abdominal Oncology, Clinical Oncology School of Fujian Medical University, Fujian Cancer Hospital, Fuzhou 350014, Fujian Province, China.; 6 Department of Otolaryngology Head and Neck Surgery, National Regional Medical Center, Binhai Campus of the First Affiliated Hospital, Fujian Medical University, Fuzhou 350212, Fujian Province, China.

**Keywords:** hypopharyngeal carcinoma cancer, LINC01569, miR-193a-5p, FADS1, macrophage polarization.

## Abstract

**Background:** Long non-coding RNA (lncRNA) LINC01569 plays an important role in regulating the tumor microenvironment (TME) and macrophage polarization. However, whether it participates in the progression of hypopharyngeal carcinoma by regulating the TME remains unclear.

**Methods:** An online database was used to analyze clinical data. Macrophage polarization was detected using qRT-PCR and flow cytometry. *In vivo* experiments were performed using tumor-bearing nude mice. A co-culture system of hypopharyngeal carcinoma cells and macrophages was used to explore the interactions between the two cell types.

**Results:** LINC01569 enhancement was observed in hypopharyngeal carcinoma tumor-associated macrophages (TAMs). In IL4-induced M2 macrophages, the expression of LINC01569 increased, while LINC01569 expression declined significantly in LPS-induced M1 macrophages. SiRNA-mediated downregulation of LINC01569 inhibits IL4-induced M2 macrophage polarization. Using online databases and a dual-luciferase reporter, miR-193a-5p was confirmed as a potential downstream sponge of LINC01569. MiR-193a-5p expression decreased in IL4-mediated M2 macrophages, which was restored by LINC01569 downregulation. Additionally, LINC01569 inhibition-mediated blocking of M2 macrophage polarization was moderately abolished by transfection with the miR-193a-5p inhibitor. Fatty acid desaturase 1 (FADS1) was verified as a downstream target of miR-193a-5p, and LINC01569 downregulation-mediated inhibition of FADS1 was blocked by miR-193a-5p mimics. Importantly, LINC01569 downregulation-mediated decline in M2 macrophage polarization was abolished by miR-193a-5p mimics, which was further reversed by FADS1 knockdown. Implantation of a mixture of FaDu cells and IL4-induced macrophages promoted tumor growth and proliferation, which were abrogated by the knockdown of LINC01569 in macrophages. Using an in co-culture system of FaDu cells and macrophages *in vitro*, M2 macrophage-regulated cell growth and apoptosis of FaDu cells were found to be mediated by the LINC01569/miR-193a-5p signaling axis.

**Conclusion:** LINC01569 is highly expressed in the TAMs of hypopharyngeal carcinoma. LINC01569 downregulation restrains macrophages from polarizing toward M2 through the miR-193a-5p/FADS1 signaling axis, thereby helping tumor cells escape inherent immune surveillance and promoting the occurrence and development of hypopharyngeal carcinoma.

## Introduction

Hypopharyngeal carcinoma is usually a squamous cell carcinoma and has the most aggressive and worst prognosis, accounting for 5-15% of all head and neck cancers 1-3. Most patients with hypopharyngeal carcinoma are asymptomatic in the early stages and diagnosed in the late stage 4. Currently, hypopharyngeal carcinoma is usually treated by surgery or radiotherapy 5. However, owing to late diagnosis and tumor metastasis, the overall five-year survival rate of patients with hypopharyngeal carcinoma is approximately 30-35% 6. Therefore, it is important to understand the pathogenesis of hypopharyngeal carcinoma and the molecular mechanisms involved in its progression and therapeutic response.

The development and progression of cancer are affected by the tumor microenvironment (TME) 7. Many studies have shown that TME affects the occurrence, metastasis, diagnosis, and treatment of hypopharyngeal carcinoma 8-10. Tumor-associated macrophages (TAMs) are the main components of the TME, widely present in tumors, and are generally considered to be involved in tumorigenesis, progression, angiogenesis, and metastasis 11, 12. Activated macrophages are generally divided into two types: M1 and M2. M1 macrophages exhibit pro-inflammatory properties through highly efficient antigen presentation 13, 14, whereas M2 macrophages have anti-inflammatory properties owing to their poor ability to present antigens and are related to immune tolerance 15, 16. Studies have shown that increased expression of astrocyte-elevated gene-1 in macrophages promotes the invasion of hypopharyngeal carcinoma 17. Dihydroartemisinin (DHA) can be used to inhibit the polarization of macrophages in the TME to prevent the progression and metastasis of hypopharyngeal carcinoma 18. This suggests that macrophages play an important role in the progression of hypopharyngeal carcinoma.

Long non-coding RNAs (lncRNAs) play an important role in regulating various cell activities through a variety of mechanisms and participate in the occurrence and development of tumors 19-21. In recent years, emerging evidence has shown the influence of lncRNAs on the TME 22, 23. LncRNA cox-2 inhibits hepatocellular carcinoma (HCC) tumor growth and metastasis by inhibiting the polarization of M2 macrophages, which also suggests that lncRNAs are key regulators of macrophage polarization 24. LncRNAs can compete with endogenous RNA (ceRNAs) and microRNAs (miRNAs) to regulate the expression of downstream protein-coding genes 25-27. LncRNAs can target miRNAs through a ceRNA mechanism to regulate macrophage polarization. LINC00662 upregulates the expression and secretion of WNT3A through a ceRNA mechanism to promote HCC progression and M2 macrophage polarization 28. Recent studies have shown that lipid metabolism events are often accompanied by macrophage polarization 29, 30. However, it remains unclear whether lncRNAs affect macrophage polarization by regulating lipid metabolism, thereby affecting HCC progression of hypopharyngeal carcinoma.

This study will clarify how lncRNA LINC01569 affects the polarization properties of macrophages and elucidate the underlying mechanism for LINC01569-mediated M2 macrophage polarization through *in vitro* and *in vivo* experiments. The results of this study will lay the foundation for mining LINC01569, which mediates hypopharyngeal carcinoma tumorigenesis, and provide a new strategy for targeting LINC01569 to treat hypopharyngeal carcinoma.

## Materials and methods

### Data collection and analysis

Differential expression in normal human hypopharyngeal carcinoma tissues and different molecular subtypes were analyzed using the Gene Expression Profiling Interactive Analysis (GEPIA) version 2 database 31. The association between LINC01569 and miR-193a-5p expression or between miR-193a-5p and fatty acid desaturase 1 (FADS1) was assessed using the Encyclopedia of RNA Interactomes (ENCORI) Starbase 32. The binding of miR-193a-5p to LINC01569 was monitored using ENCORI and lncRNASNP online databases, and the interaction between FADS1 and miR-193a-5p was determined using TargetScan, miRDB, ENCORI, and miRWalk. Patients with hypopharyngeal carcinoma (n=6) were enrolled from Fujian Cancer Hospital between 2017 and 2021. The inclusion criteria were as follows: (1) pathological diagnosis of hypopharyngeal squamous cell carcinoma; (2) preoperative cervical ultrasound, enhanced CT, or MRI examination; (3) complete admission and follow-up data; and (4) the patient underwent laryngopharyngectomy for the first time. The exclusion criteria were as follows: (1) preoperative radiotherapy, chemotherapy, or other anti-tumor targeted therapy; (2) combined with other types of carcinoma; and (3) distant metastasis of the tumor.

### Cell culture and cell transfection

Human oral epithelial keratinocytes (HOKs) and human head and neck cancer cell lines FaDu, SCC4, TU212, and Hep-2 were obtained from the American Type Culture Collection (Manassas, VA, US). Murine macrophage RAW264.7 and human monocytic leukemia cells (THP-1) were purchased from the Cell Line Bank of the Chinese Academy of Sciences. All cells were cultured and maintained in Dulbecco's modified Eagle's medium (DMEM; Gibco, USA) containing 10% fetal bovine serum (FBS; C0232, Gibco, USA), 100 U/mL penicillin, and 100 ng/mL streptomycin (Beyotime, China) at 37 °C in 5% CO_2_. Before the experiment, THP-1 cells were treated with 100 ng/ml phorbol myristate acetate (PMA) (Sigma-Aldrich) for 48 h for differentiation into macrophages. Then, the cells were washed three times with serum-free medium to remove all traces of PMA and incubated in full DMEM for another 48 h before subsequent experiments. Raw246.7- and PMA-exposed THP-1 cells were treated with either IL4 (10 ng/mL) or LPS (10 ng/mL) for 72 h to induce the two cell types to polarize toward the M2 phenotype 33, 34. The siRNAs targeting LINC01569 or FADS1 were obtained from RiboBio Corporation (Guangzhou, China). MiR-193a-5p inhibitors/mimics and corresponding negative control miRNAs were purchased from GenePharma Co., Ltd. (Shanghai, China). For cell transfection, 100 nM siRNA LINC01569, miRNA inhibitor, or FADS1 siRNA was transfected into macrophages for 48 h with HiPerFect transfection reagent (Qiagen, USA) according to the manufacturer's instructions. A co-culture model was constructed as follows: FaDu cells were plated in the lower chamber, and THP-1 cells were plated in the upper chamber. After 48 h, the FaDu cells were harvested for subsequent experiments. All experiments were repeated in triplicates.

### Quantitative real-time polymerase chain reaction (qRT-PCR) assay

Total RNA from RAW264.7 and THP-1 cells were extracted with TRIzol reagent (Invitrogen, USA). Total miRNA was extracted from tissues and cells and quantified using the miRNeasy Serum/Plasma Kit (QIAGEN, Germany). cDNA was synthesized from the total RNA (1000 ng) using RevertAid first-strand cDNA (Fermentas; Thermo Fisher Scientific, Inc.) or a TaqMan microRNA reverse transcription kit (Applied Biosystems; Thermo Fisher Scientific, Inc.). All reactions were performed in triplicate using a SYBR Green PCR kit (Qiagen, USA) according to the manufacturer's instructions. The primer sequences used for qRT-PCR (RiboBio) are listed in **Table 1**. All statistical analyses were normalized to the internal controls U6, GAPDH, andβ-actin.

### Luciferase report test

The binding and mutant sequences of FADS1-WT, FADS1-MUT, LINC01569-WT, and LINC01569-MUT were cloned into pmirGLO dual-luciferase vectors (GenePharma, Shanghai, China). 293T cells were seeded on a 48-well plate, cultured for 24 h, and then co-transfected with wild-type or mutant LINC01569 3′-UTR-Lucfirefly luciferase constructs and miR-193a-5p mimics using lipofectamine 2000 reagent. Similarly, to analyze the binding between miR-193a-5p and FADS1, 293T cells, which were seeded on a 48-well plate for 24 h, were co-transfected with the FADS1-3'UTR reporter plasmid or the mutated type and miR-193a-5p mimics. Luciferase activity was detected at 48 h using the dual-luciferase Reporter Assay System (Beyotime), and absorbance was measured at 460 nm. Each experiment was repeated at least thrice.

### RNA pull-down assay

A biotin-labeled miR-193a-5p probe (Sangon Biotech, Shanghai, China) or a negative control probe (Sangon Biotech) was incubated with magnetic beads (Thermo Fisher, 88817). Subsequently, the lysate of Raw264.7 cells that were induced by IL-4 were incubated with miR-193a-5p or oligo probes at 4 ℃ overnight and then washed by adding the appropriate buffer. The beads bound to the RNA complexes were separated and extracted using a Pierce Magnetic RNA Protein Pull-Down Kit (Thermo Fisher Scientific) for real-time PCR. Finally, RT-qPCR was used to detect the specificity of RNA in the precipitates.

### Animal models

BALB/c mice (4-5 weeks old) were purchased from Charles River Laboratories (Beijing, China). To evaluate the effect of LINC01569 on the growth of macrophages in hypopharyngeal carcinoma, mice were randomly selected and divided into three groups (five animals per condition). The groups were as follows: FaDu+THP1-control, FaDu+THP1+IL4, FaDu+THP1-siCtrl+IL4, and FaDu+THP1-LINC01569 siRNA+IL4. THP-1 cells were pretreated with or without 100 ng/mL PMA for 24 h. Next, FaDu cells (4 × 10^6^) mixed with conditioned macrophages (THP1 cells, 1 × 10^6^) were injected subcutaneously into nude mice to construct a tumor-bearing model in nude mice. The mice were maintained for 4 weeks at room temperature (22-25 °C), normal circadian rhythm fed with a regular sterile chow diet, and provided water ad libitum. From day 10 after the initial injection, tumor growth in the mice was monitored every 2 days, and the mice were sacrificed on day 22 after inoculation. Tumor volume was calculated using the following formula: 0.5 × length × width^2^. Finally, the tumor tissues were collected and weighed. All experimental procedures were approved by the Animal Welfare Committee of the Research Organization of the First Affiliated Hospital of Fujian Medical University.

### Flow cytometry

RAW264.7 and THP-1 cells were collected with a scraper, 3% BSA was used to block cells, and then cells were incubated with FITC-conjugated CD206 and CD86 for 30 min on ice in staining buffer according to the manufacturer's protocol. Data were analyzed using FlowJo software (Tree Star Inc., Ashland, OR, USA). To measure apoptosis, FaDu cells were collected in 6-well plates 48 h after culture. Subsequently, the FaDu cells were digested and centrifuged at 300 × *g* for 5 min. Cells were resuspended in ice-cold PBS and then centrifuged at 4 °C. After removal of PBS, 5 μL Annexin V-FIFC and 100 μL propidium iodide were added for 30 min incubation in the dark. The cells were analyzed by flow cytometry (EPICS, XL-4, Beckman, CA, USA) within 1 h. All experiments were repeated at least thrice.

### CCK-8

THP-1 cells were transfected with LINC01569 or an miR-193a-5p inhibitor. Subsequently, FaDu cells were seeded in the lower chamber, and THP-1 cells were seeded in the upper chamber. The cells were co-cultured for 24, 48, 72, and 96 h in a Transwell system (BD Biosciences, San Jose, CA, USA). At the indicated time point (24, 48, 72 and 96 h), 10 μL CCK-8 reagent (Beijing Dingguo Biotechnology Co., Ltd., China) was added to each well and incubated at 37 ℃ for an additional 2 h. A microplate reader (Eemak; Bio-Rad) was used to measure absorbance at the wavelength of 450 nm.

### Immunohistochemistry (IHC)

Tumor tissues were fixed in 4% paraformaldehyde for >24 h and embedded in paraffin. Then, 5 μm tissue sections were cut according to standard protocols. First, tissue slices were dewaxed in xylene and hydrated in graded ethanol solutions. Then, 0.3% H_2_O_2_ was added to the slices in an autoclave and incubated for 5-20 min to prevent antigen retrieval. After blocking with 1% bovine serum albumin for 1 h, the slices were incubated with antibodies against Ki67 (ab15580, Abcam, UK) overnight. The following day, after 1 h of incubation with the secondary antibody (PV-6001; ZSGB-BIO), a DAB detection kit (ZLI-9018; ZSGBBIO) was used for color development. IHC analysis results were obtained using a digital slide scanning system (Pannoramic Scan, 3DHISTECH, Ltd.).

### Western blot analysis

Protein from macrophages, THP-1 cells, FaDu cells, and tumor tissues were dissolved in radioimmunoprecipitation assay buffer (Applygen, Beijing, China). Equal amounts of protein lysates were loaded onto a 10% SDS-PAGE gel for electrophoresis and transferred to a PVDF membrane. After blocking with 5% nonfat milk, the membranes were incubated overnight at 4 °C with primary antibodies against FADS1 (ab126706, Abcam, UK), FADS2 (ab232898, Abcam, UK), cyclin B1 (ab32053, Abcam, UK), CDK1 (ab210008, Abcam, UK), Bcl-2 (ab32124, Abcam, UK), and BAX (ab32503, Abcam, UK). The next day, the membranes were then incubated with secondary antibodies (dilution 1:5000) for 1 h at 25 °C. After washing three times with TBST for 10 min, the blots were visualized using a chemiluminescence western blotting system and quantified using ImageJ software (version 1.8.0, National Institutes of Health, USA).

### Statistical analysis

All experimental assays were repeated at least thrice. All statistical data were analyzed using GraphPad software (version 9.0; La Jolla, CA, USA). Comparisons between different groups were performed using the unpaired Student's two-tailed *t*-test or one-way ANOVA. All values are reported as the mean ± SD from three independent experiments, and the probability value (p < 0.05) was considered statistically significant.

## Results

### Highly expressed LINC01569 is observed in M2 macrophages

**Figure 1:**
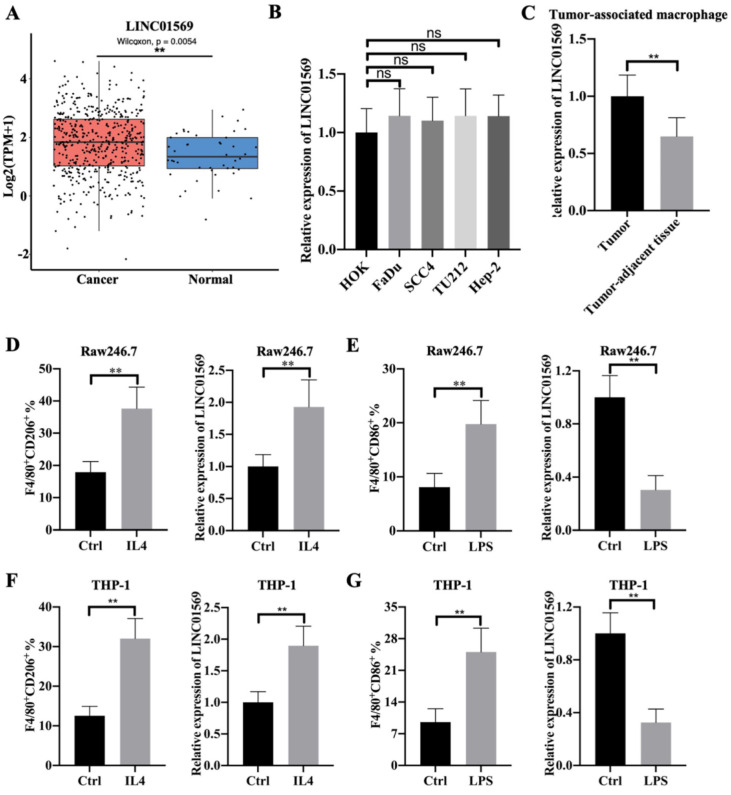
** Identification of LINC01569 as a potential mediator of M2 macrophage polarization. (A)** Differential expression of LINC01569 in head and neck squamous cell carcinoma (HNSCC) and HNSCC-free specimens was determined using the GEPIA 2 database. **(B)** LINC01569 expression in human oral epithelial keratinocytes (HOK), hypopharyngeal cancer cells (FaDu), and other HNSCC cells (SCC4, TU212 and Hep-2 cells) as measured by qRT-PCR. **(C)** The expression of LINC01569 in tumor-associated macrophages (TAMs) and adjacent tumor-associated macrophages (n=6) as determined by qRT-PCR. **(D and E)** Raw246.7 cells were treated with IL4 or LPS for 72 h. Subsequently, flow-cytometric analysis was performed to analyze the percentage of F4/80^+^CD206^+^ or F4/80^+^CD86^+^ cells in Raw246.7 cells (D), and qRT-PCR analysis was performed to analyze the RNA level of LINC01569 in Raw246.7 cells (E). A two-tailed *t*-test was used for the statistical analysis. The bar indicates the SD values. **P<0.01. **(F and G)** THP-1 cells were treated with IL4 or LPS for 72 h. Flow-cytometric analysis was performed to analyze the percentage of F4/80^+^CD206^+^ or F4/80^+^CD86^+^ cells in THP-1 cells, and qRT-PCR analysis was performed to analyze the RNA level of LINC01569 in THP-1 cells. A two-tailed *t*-test was used for the statistical analysis. The bar indicates the SD values. **P<0.01.

To investigate the role of LINC01569 in hypopharyngeal carcinoma, we first examined the expression level of LINC01569 in hypopharyngeal carcinoma and found a significant increase in LINC01569 expression in human hypopharyngeal carcinoma tissues compared to that in normal tissues using the GEPIA 2 online database **(Fig. 1A)**. However, there was no difference in LINC01569 expression between oral epithelial keratinocyte HOK cells, hypopharyngeal carcinoma FaDu cells, and other head and neck squamous cell carcinoma cell lines, including SCC4, TU212, and Hep-2 **(Fig. 1B)**. Interestingly, LINC01569 enhancement was observed in the TAMs of hypopharyngeal carcinoma tissues, in contrast to the corresponding tumor-adjacent tissue** (Fig. 1C)**. Therefore, the differential expression of LINC01569 in hypopharyngeal carcinoma may depend on the abnormal expression of LINC01569 in TAMs.

Flow-cytometric analysis showed that IL4 treatment led to an increase in the percentage of F4/80^+^CD206^+^ cells in Raw246.7 and THP-1 cells, which indicated the successful induction of M2 macrophage polarization** (Fig. 1D and F)**. In IL4-mediated M2 macrophages, the level of LINC01569 was significantly increased compared to that in the control group **(Fig. 1D and F)**. To further explore the role of LINC01569 in macrophage polarization, LPS was used to treat Raw246.7 and THP-1 cells to induce M1 macrophage polarization. Flow cytometry analysis showed that LPS administration increased the percentage of F4/80^+^CD86^+^ cells, confirming the induction of M1 macrophage polarization **(Fig. 1E and G)**. However, a decline in LINC01569 expression was observed in LPS-mediated M1 macrophages compared to that in the control group **(Fig. 1E and G)**. Taken together, these data suggest that LINC01569 expression changes during M2 macrophage polarization.

### LINC01569 knockdown attenuates IL4-induced M2 macrophage polarization

**Figure 2:**
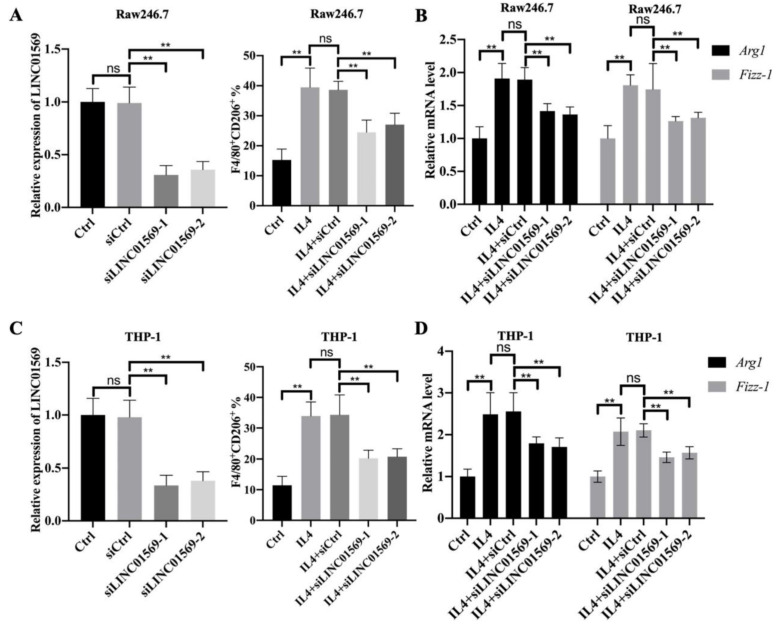
** Effects of LINC01569 on M2 macrophage polarization. (A and C)** Two siRNAs targeting LINC01569 were constructed. THP-1 and RAW264.7 cells were transfected with two siRNAs of LINC01569 and negative control siRNA for 48 h and then treated with IL4 or LPS for 72 h. The knockdown efficiency was confirmed in THP-1 and RAW264.7 cells using qRT-PCR analysis (A and C: left). Flow-cytometric analysis was performed to analyze the percentage of CD206^+^ cells (A and C: right). **(B and D)** mRNA of M2 polarization marker genes including *Arg1* and *Fizz-1* in THP-1 and RAW264.7 cells was quantified by qRT-PCR analysis. **P<0.01.

Given that LINC01569 is upregulated in IL4-exposed macrophages, the role of LINC01569 in M2 macrophage polarization was further investigated. Firstly, the expression of LINC01569 was knocked down in Raw246.7 and THP-1 cells that were transfected with LINC01569 siRNA. qRT-PCR analysis indicated that transfection with siRNAs targeting LINC01569 significantly inhibited the expression of LINC01569 compared to control cells transfected with negative control siRNA **(Fig. 2A and C)**. Once LINC01569 was knocked down, IL4-induced M2 macrophage polarization was abolished, which was reflected in the decrease in the percentage of F4/80^+^CD206^+^ cells in both Raw246.7 and THP-1 cells by flow-cytometric analysis, compared to that in IL4-exposed macrophages **(Fig. 2A and C)**. Relative marker genes for M2 macrophage polarization were examined to confirm our results. qRT-PCR analysis showed that the levels of *Arg1* and *Fizz-1* notably increased with the treatment of IL4 compared to those of the control group in Raw246.7 and THP-1 cells, which were obviously inhibited by LINC01569 knockdown **(Fig. 2B and D)**. Thus, these data indicate that LINC01569 knockdown restrained IL4-mediated M2 macrophage polarization.

### LINC01569 is a potential sponge of miR-193a-5p

**Figure 3:**
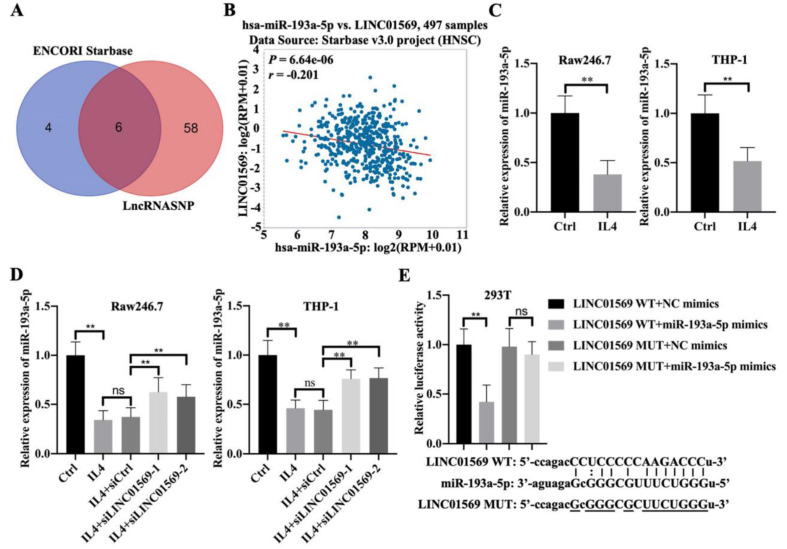
** LINC01569 sequesters miR-193a-5p in macrophages. (A)** Potential downstream target microRNAs were analyzed by combining the ENCORI and LncRNASNP online database.** (B)** The association between LINC01569 and miR-193a-5p expression in hypopharyngeal carcinoma specimens (n=497) was monitored using the ENCORI online database. R: Pearson coefficient. **(C)** THP-1 and RAW264.7 cells were pretreated with IL4 for 72 h. qRT-PCR analysis was performed to analyze the mRNA levels of miR-193a-5p in THP-1 and RAW264.7 cells. **(D)** THP-1 and RAW264.7 cells were transfected with siLINC01569-1 or siLINC01569-2 followed by the treatment of IL4 or not. Subsequently, the miR-193a-5p level was determined by qRT-PCR. **(E)** The binding sequence between LINC01569 and miR-193a-5p was obtained using ENCORI Starbase. After transfection with LINC01569-WT and LINC01569-MUT reporter plasmids in the presence of NC mimics or miR-193a-5p mimics, luciferase activity was determined using the corresponding kits. ** indicated cells transfected with LINC01569 WT and cells transfected miR-193a-5p mimic vs. LINC01569 WT and NC mimic. WT, wild-type; MUT, mutation. **P<0.01.

To better understand the mechanism by which LINC01569 regulates the progression of hypopharyngeal carcinoma, potential binding partners of LINC01569 were screened. Here, six potential target genes, hsa-miR-381-3p, hsa-miR-1323, hsa-miR-548o-3p, hsa-miR-103a-3p, hsa-miR-193a-5p, and hsa-miR-107, were screened as candidates for LINC01569 using the ENCORI and lncRNASNP online databases **(Fig. 3A)**. Because of the significant negative association between miR-193a-5p and LINC01569 in hypopharyngeal carcinoma tissues, miR-193a-5p was selected for subsequent experiments **(Fig. 3B)**. There was no significant correlation between the expression of the other five miRNAs and LINC01569 in hypopharyngeal carcinoma. In IL4-induced M2 macrophages, a decrease in miR-193a-5p was observed compared to control Raw246.7 and THP-1 cells **(Fig. 3C)**. However, the IL4-mediated decrease in miR-193a-5p in Raw246.7 and THP-1 cells was moderately restored by the transfection of siRNA targeting LINC01569 **(Fig. 3D)**. A luciferase reporter assay was performed to confirm the direct relationship between miR-193a-5p and LINC01569 expression. The luciferase activity of the LINC01569 WT reporter was notably abrogated by transfection with miR-193a-5p mimic, whereas miR-193a-5p mimic did not affect the luciferase activity of cells transfected with the LINC01569 reporter plasmid with a site-directed mutation** (Fig. 3E)**. These data provided evidence that LINC01569 acts as a sponge for miR-193a-5p.

### IL4-induced M2 macrophage polarization depends on the LINC01569/miR-193a-5p signaling axis

**Figure 4:**
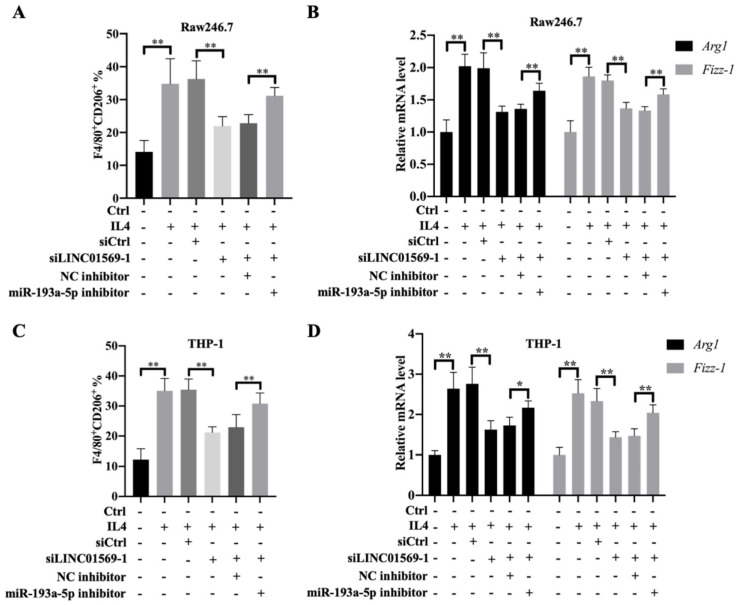
** Effect of miR-193a-5p on LINC01569 in maintaining M2 polarization of macrophages.** THP-1 and RAW264.7 cells were transfected with LINC01569 siRNA, miR-193a-5p inhibitor, or their corresponding control siRNA/inhibitor, followed by the treatment with IL4 or not. **(A and C)** Flow-cytometric analysis was performed to analyze the percentage of F4/80^+^CD206^+^ cells in THP-1 and RAW264.7 cells. **(B and D)** mRNA of M2 polarization marker genes including *Arg1* and *Fizz-1* in THP-1 and RAW264.7 cells was quantified by qRT-PCR analysis. *P<0.05; **P<0.01.

To further verify the effect of miR-193a-5p on M2 polarization of macrophages, Raw246.7 and THP-1 cells were transfected with LINC01569 siRNA along with a combination of LINC01569 siRNA and miR-193a-5p inhibitor. IL4 treatment significantly increased the proportion of M2 macrophages, and LINC01569 siRNA transfection abolished the promotive effects of IL4 in M2 macrophage polarization, while miR-193a-5p inhibitor management restrained the effects of LINC01569 siRNA and moderately restored the percentage of F4/80^+^CD206^+^ in Raw246.7 and THP-1 cells compared with the NC inhibitor transfection **(Fig. 4A and C)**. Marker genes for M2 macrophage polarization were also examined. qRT-PCR analysis showed that the relative mRNA expression of *Arg1* and *Fizz-1* increased with the treatment of IL4 compared to that in the control group, which was reversed by LINC01569 knockdown. However, the levels of Arg1 and Fizz-1 were restored by the addition of miR-193a-5p inhibitor in Raw246.7 and THP-1 cells **(Fig. 4B and D)**. These data demonstrate that IL4-induced M2 macrophage polarization is regulated by the LINC01569/miR-193a-5p signaling axis.

### Fatty acid desaturase 1 (FADS1) is a downstream target gene of miR-193a-5p

**Figure 5:**
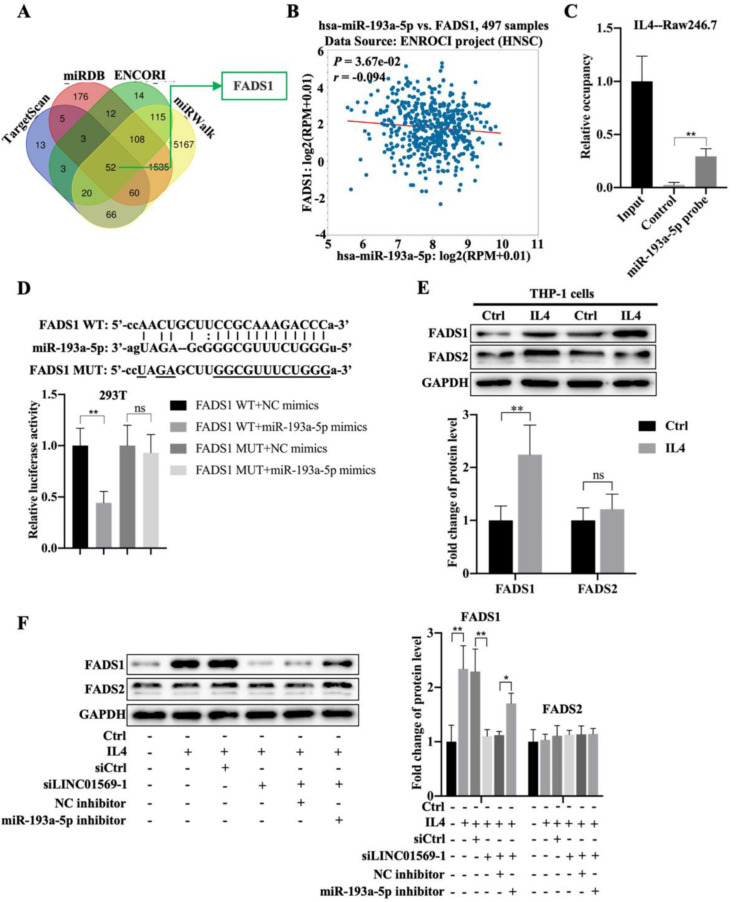
** Association between miR-193a-5p and fatty acid desaturase 1 (FADS1) during M2 polarization of macrophages. (A)** The downstream targets of miR-193a-5p as analyzed through TargetScan, miRDB, ENCORI, and miRWalk online databases. **(B)** The association between FADS1 and miR-193a-5p expression in hypopharyngeal carcinoma specimens was analyzed by SPSS using ENCORI online databases. R: Pearson coefficient. **(C)** RNA pull-down was used to present the binding between miR-193a-5p as fold enrichment. **(D)** Dual-luciferase analysis was performed when THP-1 cells were co-transfected with FADS1-wt and miR-539-5p mimic or FADS1-mut and miR-539-5p mimic. The activity of luciferase was detected. **(E)** THP-1 cells were treated with IL4 for 72 h. Western blotting was performed to detect the protein levels of FADS1 and FADS2. **(F)** THP-1 cells were transfected with LINC01569 siRNA or the combination of LINC01569 siRNA and miR-193a-5p inhibitor for 72 h. Western blotting was performed to detect the protein levels of FADS1 and FADS2. *P<0.05; **P<0.01.

Next, we investigated the downstream targets of miR-193a-5p. Using online databases such as TargetScan, miRDB, ENCORI, and miRWalk, fifty-two potential downstream targets were screened **(Fig. 5A)**. Based on the literature and our preliminary results, FADS1 was selected as a candidate target gene of miR-193a-5p. FADS1 expression was elevated in hypopharyngeal carcinoma tissues compared to adjacent normal tissues **(Fig. S1)**. Importantly, miR-193a-5p expression negatively correlated with FADS1 expression in hypopharyngeal carcinoma **(Fig. 5B)**. Pull-down experiments further confirmed the direct binding of miR-193a-5p to FADS1** (Fig. 5C)**. The relative luciferase activity of the vector expressing WT-FADS1 was significantly decreased by co-transfection with miR-193a-5p mimics; however, miR-193a-5p mimic transfection did not affect the relative luciferase activity of the vector expressing MUT-FADS1 **(Fig. 5D)**. However, the expression of FADS1 during macrophage polarization requires further investigation. In IL4-treated THP-1 cells, the level of FADS1 increased by adding IL4, while the expression of FADS2 showed no obvious change**s (Fig. 5E)**. Importantly, the IL4-induced increase in FADS1 expression was abolished by the transfection of LINC01569 siRNAs, which was further reversed by the effect of miR-193a-5p inhibitor in THP-1 cells **(Fig. 5F)**. The expression of FADS2 was not affected by the LINC01569 or miR-193a-5p knockdown **(Fig. 5F)**. These results suggest that miR-193a-5p directly targets FADS1, and FADS1 is regulated by the LINC01569/miR-193a-5p signaling axis during the progression of M2 macrophage polarization.

### LINC01569 induces M2 polarization of macrophages through miR-193a-5p/FADS1

**Figure 6:**
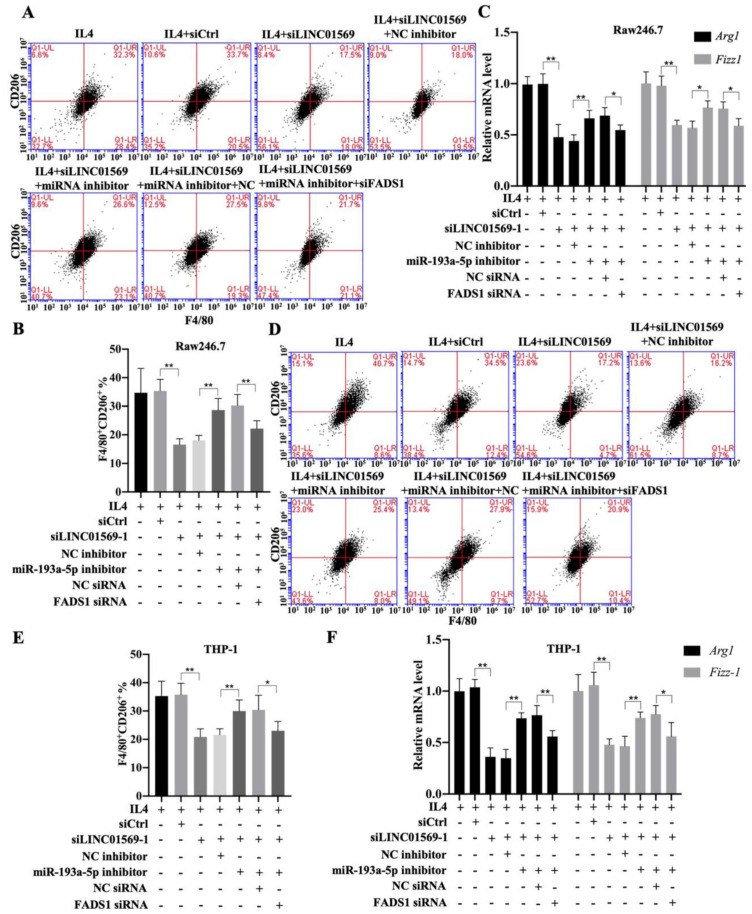
**Effects of LINC01569/miR-193a-5p/FADS1 on M2 polarization of macrophages.** THP-1 and RAW264.7 cells were transfected with LINC01569 siRNA, miR-193a-5p inhibitor, FADS1 siRNA, or their corresponding control siRNA/inhibitor, followed by the treatment with IL4 or not. **(A and D)** Flow-cytometric analysis was performed to analyze the percentage of F4/80^+^CD206^+^ cells in THP-1 and RAW264.7 cells. **(B and E)** Quantitative analysis of flow-cytometric results in THP-1 and RAW264.7 cells.** (C and F)** mRNA of M2 polarization marker genes including *Arg1* and *Fizz-1* in THP-1 and RAW264.7 cells was quantified by qRT-PCR analysis. *P<0.05; **P<0.01.

Subsequently, the effect of FADS1 on LINC01569-regulated M2 polarization was investigated. As shown in Figure 6, the percentage of F4/80^+^CD206^+^ cells in IL4-treated Raw246.7 and THP-1 cells decreased with LINC01569 siRNA transfection. After co-transfection with LINC01569 siRNA and miR-193a-5p inhibitor, the percentage of F4/80^+^CD206^+^ cells in IL4-treated Raw246.7 and THP-1 cells was restored compared to that obtained from LINC01569 siRNA transfection alone **(Fig. 6A-6B and 6D-6E)**. However, miR-193a-5p inhibitor-mediated resistance to LINC01569 siRNA was reversed by knockdown of FADS1 in both Raw246.7 and THP-1 cells **(Fig. 6A-6B and 6D-6E)**. Meanwhile, IL4-mediated elevation of Arg1 and Fizz-1 expression was counteracted by LINC01569 knockdown, which was reversed by the addition of the miR-193a-5p inhibitor to Raw246.7 and THP-1 cells **(Fig. 6C and 6F)**. However, transfection with FADS1 siRNA neutralized the effects of the miR-193a-5p inhibitor and played a role similar to that of LINC01569 siRNA in IL4-induced M2 macrophage polarization **(Fig. 6C and 6F)**. M2 macrophage polarization may be impaired by regulation of the LINC01569/miR-193a-5p/FADS1 signaling axis.

### LINC01569 loss protects against M2 macrophages-driven tumorigenesis

**Figure 7:**
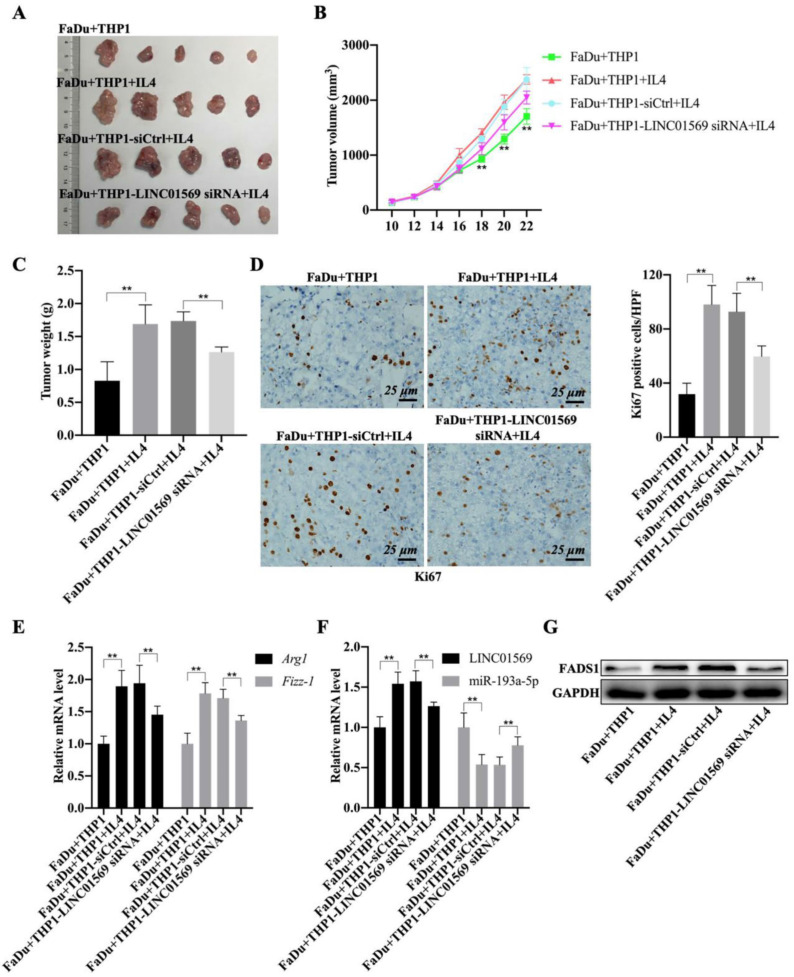
** Effects of LINC01569 knockdown on tumor growth. (A)** FaDu cells with THP-1 cells (control, IL4-activated, IL4-activated+siNC, and IL4-activated+LINC01569 siRNA) were inoculate subcutaneously in mice (n = 5) at the endpoint of the mice study. The tumor images of mice as photographed. **(B)** Tumor volume was measured with 2-day intervals on day 10 after implantation. **(C)** The body weight of mice was measured at the endpoint of the mice study. **(D)** Immunohistochemistry analysis of the expression of Ki67 in the xenograft tumor tissues. Scale bar = 50 μm. Relative quantitative analysis of Ki67-positive cell number as showed on the right. **(E)** qRT-PCR analysis was performed to measure the mRNA level of M2 polarization markers including *Arg1* and *Fizz-1* in tumor tissues. **(F)** The expression levels of LINC01569 and miR-193a-5p mRNA were assessed by qRT-PCR in tumor tissues. **(G)** The protein level of FADS1 was tested via western blot. **P<0.01.

Given the role of M2 macrophages in promoting tumorigenesis, the effect of LINC01569 on the crosstalk between functional macrophages and tumor cells was investigated using tumor-bearing mice. As expected, the mixture of IL4-activated macrophages and FaDu cells showed greater tumor size, faster growth rate, and higher weight of mouse tumors than the control group (FaDu cells and M0 macrophages) **(Fig. 7A-C)**. Conversely, LINC01569 knockdown in IL4-exposed macrophages significantly diminished the size, volume, and weight of mouse tumors compared to the activated group (IL4-activated macrophages and FaDu cells) **(Fig. 7A-C)**. Furthermore, Ki67 expression was measured in subcutaneous tumors using IHC staining.

The staining results revealed that the activated macrophages (FaDu+THP1+IL4) notably increased the expression of Ki67 in tumor tissues, and the knockdown of LINC01569 in THP1 cells (FaDu+THP1-LINC01569 siRNA+IL4) abrogated the effects of activated macrophages on the expression of Ki67, which indicated that tumor growth could be reinforced by IL-4-exposed THP1 cells, and the protumour effect of M2 macrophages could be reversed by treatment with LINC01569 siRNA **(Fig. 7D)**. Meanwhile, IL4-mediated macrophage activation significantly increased the mRNA levels of Arg1 and Fizz-1 and inhibited the expression of iNOS compared to the control group, which was blocked by transfection of LINC01569 siRNA in THP1 cells in the presence of IL4 **(Fig. 7E and Fig. S2)**. Moreover, the expression of LINC01569 and FADS1 was elevated, and that of miR-193a-5p was mitigated in the activated group (IL4-activated macrophages and FaDu cells), which was neutralized by LINC01569 removal in IL4-exposed THP1 cells **(Fig. 7F-G and Fig. S3)**. Overall, LINC01569 restrained M2 macrophage-mediated tumor growth in hypopharyngeal carcinoma.

### LncRNA LINC01569/miR-193a-5p/FADS1 in macrophages promotes the growth and apoptosis of hypopharyngeal carcinoma cells

**Figure 8:**
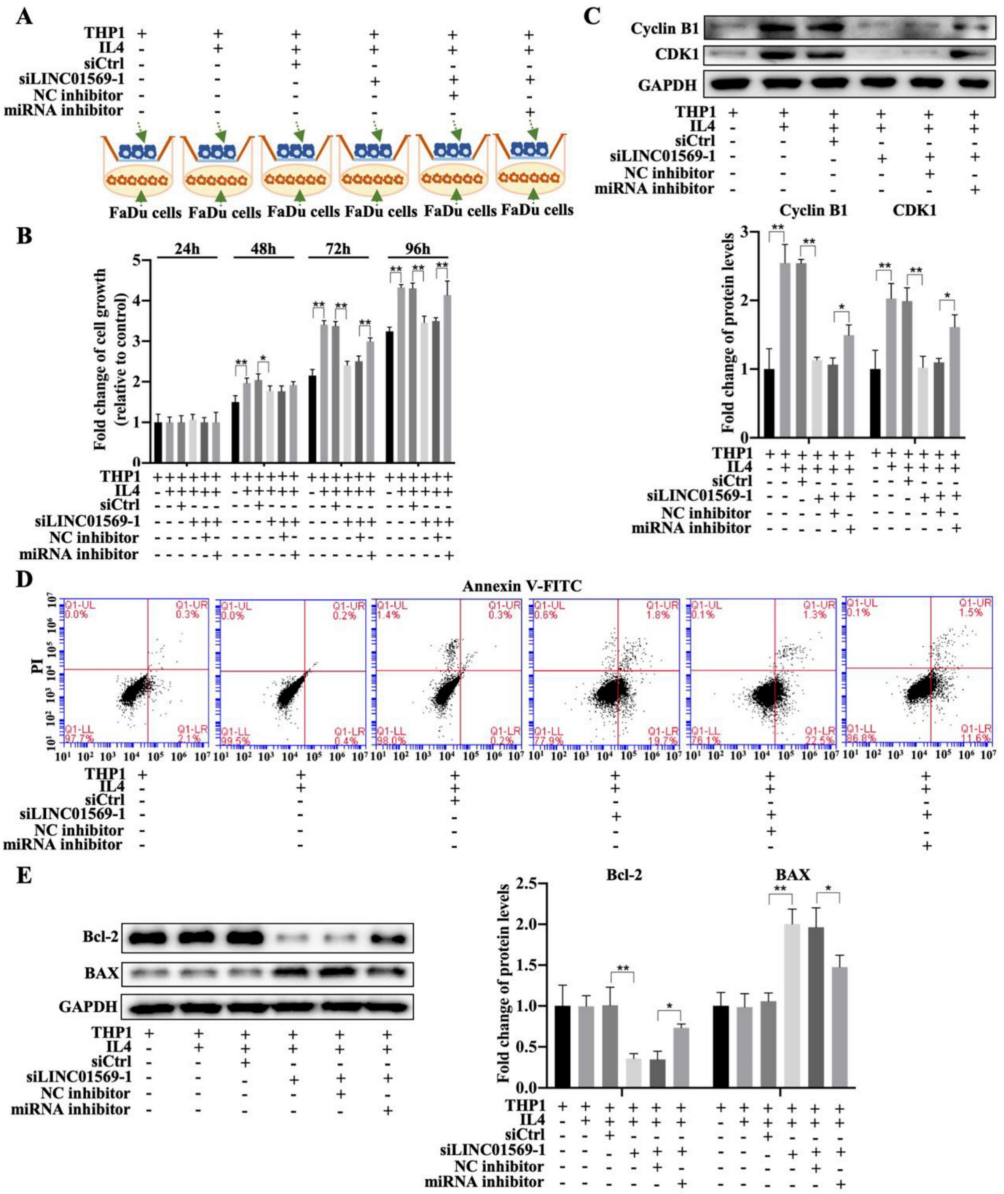
** Effects of LINC01569/miR-193a-5p in macrophages on cell viability and apoptosis of FaDu cells. (A)** Treatment chart of co-culture of FaDu Cells and THP-1 cells that were pre-transfected with LINC01569 siRNA alone or the combination with LINC01569 siRNA and miR-193a-5p inhibitor. **(B)** The CCK-8 assay was used to determine cell proliferative capacity after transfection at 24, 48, 72, and 96 h.** (C)** Western blotting was employed to test protein expression of Cyclin B1 and CDK1 after transfection at 48 h. **(D)** The apoptotic rate was evaluated by flow cytometry. **(F)** Western blot was applied to detect the protein levels of Bax and Bcl-2. *P<0.05; **P<0.01.

To further elucidate the effect of LINC01569/miR-193a-5p/FADS1 in macrophages on hypopharyngeal carcinoma cell growth, a co-culture of macrophages and hypopharyngeal carcinoma cells (transfected with LINC01569 siRNA or miR-193a-5p inhibitor) was used to detect tumor cell proliferation and apoptosis combining with the Transwell apparatus **(Fig. 8A)**. The CCK-8 assay indicated that co-culture of FaDu cells and IL4-exposed macrophages increased cell viability, and LINC01569 siRNA addition in macrophages reduced the effects of M2 macrophages on FaDu cell proliferation from 48 to 96 h, while the miR-193a-5p inhibitor could reverse the anti-tumor effect of LINC01569 siRNA from 72 to 96 h **(Fig. 8B)**. Similarly, the protein expression of the proliferation-related markers Cyclin B1 and CDK1 increased along with the induction of M2 macrophage polarization, which was decreased by LINC01569 knockdown. MiR-193a-5p inhibitor transfection reversed the effects of the LINC01569 siRNA on their expression **(Fig. 8C)**. However, IL4 mediated macrophage activation and did not affect tumor cell apoptosis. Once LINC01569 was knocked down in macrophages, the proportion of apoptotic cells increased following the administration of IL4, which was abolished by transfection with the miR-193a-5p inhibitor (**Fig. 8D and Fig. S4**). The effects of LINC01569 and miR-193a-5p inhibitors on apoptosis were also reflected in the protein expression of Bcl-2 and Bax. In the presence of IL4, Bcl-2 protein levels decreased and Bax levels increased in FaDu cells when LINC01569 was silenced in macrophages in the co-culture system **(Fig. 8E)**. The changes in the expression of Bcl-2 and Bax were blocked by miR-193a-5p **inhibition (Fig. 8E)**. These results led us to conclude that the LINC01569/miR-193a-5p signaling axis in macrophages directly impacts tumor cell growth and apoptosis in hypopharyngeal carcinoma cells.

## Discussion

The TME plays an important role in the development and progression of cancer 35. Recent progress has shown that the TME affects recurrence, metastasis, and drug resistance in patients with hypopharyngeal carcinoma. A possible mechanism is that the TME avoids being recognized in the occurrence of immunosuppression by acting on immune cells in hypopharyngeal carcinoma 8, 10, 36. A related study found that the regulation promotes the invasion of hypopharyngeal carcinoma 17. In this study, we revealed that LINC01569 was upregulated in the tumor macrophages of hypopharyngeal carcinoma and promoted the occurrence of hypopharyngeal carcinoma through the LINC01569/miR-193a-5p/FADS1 axis. Therefore, this study provides a new direction for the treatment of hypopharyngeal squamous cell carcinoma.

In recent years, LncRNA was found to be closely related to the occurrence and development of tumors 37. Related research has shown that the lncRNA HOXA11-As can be used as an important prognostic marker of hypopharyngeal squamous cell carcinoma and plays an important role in tumor proliferation and apoptosis 38. MALAT1 plays a promoting role in migration and invasion of hypopharyngeal carcinoma cells (FaDu) 39. UCA1 upregulation promotes the proliferation, invasion, and survival of hypopharyngeal carcinoma cells 40. Thus, the role of lncRNAs in the development of head and neck cancers has received increasing attention. Studies have shown that LINC01569 expression is elevated in colorectal cancer. In this study, we found that LINC01569 was upregulated in head and neck tumors. However, LINC01569 expression did not differ between the hypopharyngeal cancer cells and other head and neck squamous cell carcinoma cell lines. This paradox prompted us to further distinguish the source of the differences in LINC01569 expression. Interestingly, lncRNAs have been proven to play important roles in macrophage polarization 24, 41. It has been found that knockdown of lncRNA TP73-AS1 inhibits M2 macrophage infiltration and HCC tumor growth *in vivo* 42 and LncRNA-SNHG1 promotes the growth and metastasis of breast cancer by promoting M2-like polarization of macrophages 43. We found that the expression of LINC01569 in the TAM of hypopharyngeal carcinoma was higher than that in macrophages isolated from adjacent tissues. It is possible that the difference in LINC01569 expression in hypopharyngeal carcinoma is associated with TAM abundance. It has been shown that CRNDE overexpression can promote M2 polarization of TAMs and induce tumor angiogenesis 44. In prostate cancer, high LINC00467 expression promotes cancer development by regulating M2 macrophage polarization 45. Therefore, lncRNAs play key roles in regulating M2 macrophage polarization. Our data showed that LINC01569 expression was upregulated and downregulated in the M2 and M1 macrophages, respectively. IL4-mediated M2 macrophage polarization depends on LINC01569 expression. Therefore, we conclude that LINC01569 is responsible for regulating the M2 polarization of macrophages and subsequently causes abnormal immune regulation in hypopharyngeal carcinoma.

MiR-193a-5p has carcinogenic or tumor inhibitory functions in different types of cancer, and its expression is upregulated in HCC and osteosarcoma 46, 47, whereas miR-193a-5p overexpression can inhibit the progression of rhabdomyosarcoma 48. Circular RNA hsa_circ_0001666 can sponge miR‑193a‑5p to promote papillary thyroid carcinoma progression 49. MiR-193a-5p inhibition contributes to the remission of nasopharyngeal carcinoma progression 50. Thus, miR-193a-5p is a vital regulator of head and neck tumor progression. Additionally, the function of miRNAs in the regulation of macrophage polarization has been confirmed 51. MiR-193a-5p in tumor-associated macrophage-derived exosomes can promote the development of RCC 35443741. At present, we can learn that the relationship between LINC01569 and miR-381-3p is mainly confirmed in colorectal cancer 52. In this study, we found that miR-193-5a was a potential target of LINC01569. Our experimental results showed that the miR-193-5a inhibitor significantly promoted M2 polarization. It is possible that the role of LINC01569 in regulating M2 polarization of macrophages depends on miR-193-5.

Fatty acid desaturase 1 (FADS1) is known as a key fatty acid that can inhibit the metabolism of unsaturated fatty acids 53. Studies have shown that FADS1 is highly expressed in most cancer cells, including laryngeal squamous cell carcinoma (LSCC) 54, 55. However, low expression of FADS1 indicates poor prognosis in patients with esophageal squamous cell carcinoma 56. Furthermore, knockdown of FADS1 in macrophages balanced the activation of M1 and M2 polarization of macrophages 57. Thus, the regulatory role of FADS1 in macrophage polarization may determine the development and direction of tumorigenesis. In the present study, we found that FADS1, a sponge of miR-193a-5p, was positively regulated by LINC01569 and negatively regulated by miR-193a-5p. Importantly, FADS1 knockdown reversed the miR-193a-5p-mediated increase in M2 macrophage polarization, which plays a role similar to that of LINC01569. Therefore, we concluded that LINC01569 regulates macrophage polarization through the miR-193a-5p/FADS1 signaling axis. The FADS1 products arachidonic acid (AA) and eicosapentaenoic acid (EPA) produce relevant lipid signals that regulate the inflammatory response 43, 44. Studies have shown that the expression of FADS1 increases in brain cancers of primary tumors, and the underlying mechanism for FADS1-mediated tumor progression is that FADS1 affects the cholesterol synthesis process and cell cycle arrest 58-60. FADS1-mediated metabolite production plays an essential role in cancer cell proliferation, metastasis, and the TME 58. Therefore, the lipid metabolism signaling induced by FADS1 promotes tumorigenesis. Classically activated M1 macrophages and alternatively activated M2 macrophages display distinct patterns of glucose, lipid, amino acid, and iron metabolism 61. Thus, M2 macrophage polarization regulated by LINC01569/miR-193a-5p/FADS1 may be involved in abnormal lipid metabolism signaling. In the *in vivo* study, LINC01569 knockdown in macrophages inhibited tumor growth and cell viability of hypopharyngeal cancer cells, which were reversed by the miR-193a-5p inhibitor and restored by FADS1 knockdown. Therefore, activation of the LINC01569/miR-193a-5p/FADS1 signaling axis in macrophages can accelerate M2 macrophage polarization, thereby leading to the progression of hypopharyngeal cancer. Interestingly, IL4-mediated M2 macrophage polarization did not affect apoptosis in FaDu cells but promoted cell viability. Although LINC01569 inhibition in macrophages restrained IL4-induced cell viability and increased cell apoptosis, we did not observe that LINC01569 in the LINC01569/miR-193a-5p/FADS1 signaling pathway caused excessive cell proliferation associated with cell apoptosis events. Possibly, the LINC01569/miR-193a-5p/FADS1 axis affects macrophage polarization by regulating lipid metabolism. Additionally, we need to further verify our results using large-scale clinical data. Determining whether FADS1 affects macrophage polarization by regulating lipid metabolism signaling requires further investigation. Importantly, elucidating if LINC01569 regulates other immune cells in the TME should be investigated in future studies.

## Conclusion

Our findings revealed that LINC01569 regulates macrophage polarization, resulting in the occurrence and development of hypopharyngeal carcinoma through the miR-193a-5p/FADS1 signaling axis. These data provide new directions for the diagnosis and treatment of hypopharyngeal cancer.

## Supplementary Material

Supplementary figures and table.

## Figures and Tables

**Table 1:** Primer sequences of RT-PCR.

Gene	Forward	Reverse
***LINC01569***	5'-CAGTGCCACCTTCTCTACCTGCT-3'	5'-ATCATCTTTACGGAGGTAGAGAAGAG-3'
***GAPDH***	5'-AGGTCGGTGTGAACGGATTTG-3'	5'-GGGGTCGTTGATGGCAACA-3'
***U6***	5'-CGCAAGGATGACACGCAA-3'	5'-GTGCAGGGTCCGAGGT-3'
***Arg1***	5'-TCACCTGAGCTTTGATGTCG-3'	5'-CTGAAAGGAGCCCTGTCTTG-3'
***Fizz-1***	5'-CACCTCTTCACTCGAGGGACAGTTG-3'	5'-GGTCCCAGTGCATATGGATGAGACC-3'
***iNOS***	5′-TCTTGGTCAAAGCTGTGCTC-3′	5′-CATTGCCAAACGTACTGGTC-3′
***Arg1-m***	5'-GAACCCAACTCTTGGGAAGAC-3'	5'-GGAGAAGGCGTTTGCTTAGTT-3'
***Fizz-1-m***	5'-CACCTCTTCACTCGAGGGACAGTTG-3'	5'-GGTCCCAGTGCATATGGATGAGACC-3'
***iNOS-m***	5'-CTCTACAACATCCTGGAGCAAGTG-3'	5'-ACTATGGAGCACAGCCACATTGA-3'
***β-actin-m***	5'-CTGACTGACTACCTCATGAAGATCCT-3'	5'-CTTAATGTCACGCACGATTTCC-3'
***U6-m***	5'-GCTTCGGCAGCACATATACTAAAAT-3'	5'-CGCTTCACGAATTTGCGTGTCAT-3'
***hsa-miR-193a-5p***	5'-TATATGGGTCTTTGCGGGCG-3'	5'-GTGCAGGGTCCGAGGT-3'
***Mmu-miR-193a***	5'-CCCCTAATACTGCCTGGTAATGA-3'	5'-GTGCAGGGTCCGAGGT-3'

## Abbreviations

TME: tumor microenvironment
TAM: Tumor-associated macrophage
FADS1: Fatty acid desaturase 1
LncRNAs: Long non coding RNAs
HCC: hepatocellular carcinoma
