# Supplementary material for: LncRNA LINC01569 promotes M2 macrophage polarization to accelerate hypopharyngeal carcinoma progression through the miR-193a-5p/FADS1 signaling axis
Source: J Cancer. 2023 Jun 4;14(9):1673–88. doi: 10.7150/jca.83466 (PMC10266250; doi:10.7150/jca.83466)
Supplement: Supplementary file 1 — Supplementary figures and table. [file jcav14p1673s1.pdf]

Supplementary figure legends

Figure S1

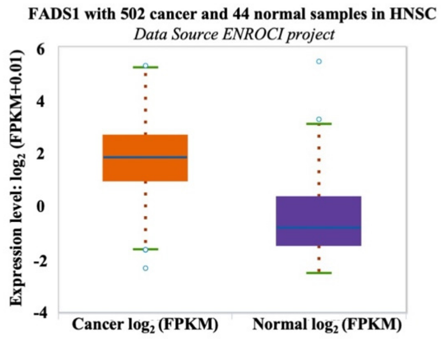

Fig. S1 FADS1 expression in normal tissues and HNSCC tissues.

Figure S2

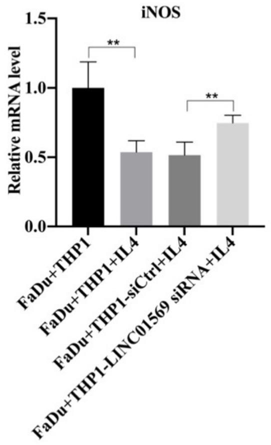

Fig. S2 Relative mRNA expression of iNOS. qRT-PCR analysis was performed to measure the mRNA level of M2 polarization marker iNOS in tumor tissues.

Figure S3

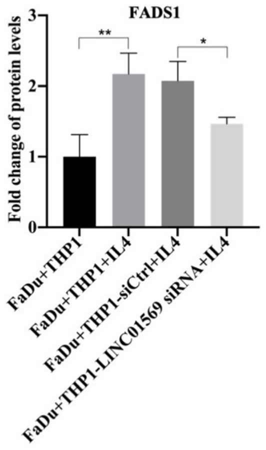

Fig. S3 The relevant quantification results. Quantification analysis of FADS1 protein expression in figure 7G. *P<0.05; **P<0.01.

Figure S4

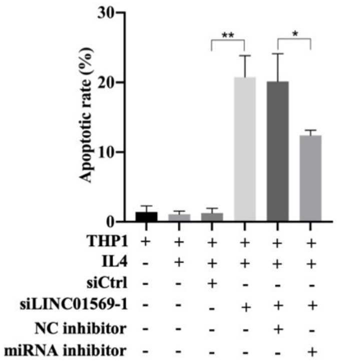

Fig. S4 The relevant quantification results of apoptotic cells. Quantification results of apoptotic rate in FaDu Cells in Figure 8D. *P<0.05; **P<0.01.

Table S1 Clinic parameters of enrolled patients.

Serial number	Gender	Age	Pathology	Therapies
1	Male	56	Poorly differentiated hypopharyngeal squamous cell carcinoma	Neoadjuvant therapy
2	Male	62	moderately differentiated hypopharyngeal squamous cell carcinoma	Neoadjuvant therapy
3	Female	58	Highly differentiated hypopharyngeal squamous cell carcinoma	Surgical excision
4	Male	49	moderately differentiated hypopharyngeal squamous cell carcinoma	Neoadjuvant therapy
5	Male	63	moderately differentiated hypopharyngeal squamous cell carcinoma	Neoadjuvant therapy
6	Female	61	Poorly differentiated hypopharyngeal squamous cell carcinoma	Neoadjuvant therapy
